# Stroke in Bangladesh: A Narrative Review of Epidemiology, Risk Factors and Acute Stroke Services

**DOI:** 10.3390/jcdd12020058

**Published:** 2025-02-05

**Authors:** Mudasar Aziz, Naznin Bipasha, Udit Gupta, Isabela V. P. Ramnarine, Jessica Redgrave, Ali N. Ali, Arshad Majid, Simon M. Bell

**Affiliations:** 1 Sheffield Institute of Translational Neuroscience, University of Sheffield, Sheffield S10 2GF, UK; mudasar.aziz@nhs.net (M.A.); udit.gupta@nhs.net (U.G.); isabela.ramnarine@nhs.net (I.V.P.R.); jessica.redgrave@nhs.net (J.R.); ali.ali9@nhs.net (A.N.A.); arshad.majid@sheffield.ac.uk (A.M.); 2 Department of Neurology, University Hospitals Southampton, Southampton SO16 6YD, UK; naznin.bipasha@nhs.net; 3 Department of Neurology, Sheffield Teaching Hospitals NHS Foundation Trust, Royal Hallamshire Hospital, Sheffield S10 2GJ, UK

**Keywords:** Bangladesh, stroke, prevalence, incidence, management, services

## Abstract

Introduction: Stroke is a major cause of mortality and disability worldwide. It is one of the foremost non-communicable diseases and the third leading cause of death in Bangladesh. Bangladesh is a developing country and as such, the prevalence, risk factors and management of the condition has some differences with more Westernised populations. In this review, we look at the epidemiology of stroke in Bangladesh and what barriers exist to improving stroke care in this country. Methods: A systematic search of the PubMed database, Mymensingh Medical Journal, Bangladesh Journal of Neuroscience and Google Scholar was conducted for articles relating to stroke in Bangladesh. Results: The incidence of stroke in Bangladesh is 3 strokes per 1000 people. The prevalence of haemorrhagic stroke is higher than in Western populations, and novel risk factors such as sitting in a squatting position and betel nut chewing increase a Bangladeshi’s stroke risk. A lack of education on stroke symptoms and a poor internal infrastructure are the main barriers to improving stroke care in this country. Conclusions: Stroke in Bangladesh is associated with traditional risk factors and non-traditional risk factors that are behaviourally ingrained into the local culture. Improved outcomes for these patients will rely on education programmes for the population, a regard for these risk factors and improving the availability of evidence-based treatments. Innovative approaches from policy decision makers to enhance equitable access to stroke services may help in providing stroke care in Bangladesh.

## 1. Introduction

Stroke is one of the leading causes of mortality and disability worldwide. Nearly 15 million strokes occur each year, resulting in 6 million deaths and 5 million cases of permanent disability [1]. Although it is declining in developed nations, the number of deaths due to stroke in developing countries is increasing [1]. According to a 2001 estimate made by the World Health Organisation (WHO), 86% of stroke-related deaths worldwide occur in people from developing countries, 40% of whom live in South Asia. In South Asia, stroke occurs a decade earlier, on average, than in the rest of the world [2]. As one of the South Asian nations considered to be a developing country, Bangladesh is experiencing a dramatic increase in non-communicable diseases of old age, such as coronary artery disease and stroke, due to changes in socio-economic conditions, urbanisation, changes in diet and an increased life expectancy [2].

Bangladesh has a diverse healthcare insurance system which is provided by four main sectors: the government, non-governmental organisations, the private sector and donor agencies [3]. Despite the public sector being heavily funded by the government, the quality of care is considered to be poor due to insufficient funding and governance. As a result, the private sector has expanded, despite it being unaffordable for a majority of the population, and either social or private insurance being uncommon [3].

Stroke is the third leading medical cause of death in Bangladesh. According to the WHO health data from 2011, around 82,039 deaths due to stroke were recorded, which represents 8.57% of the total deaths [4]. The age- and sex-standardised mortality rate for stroke in Bangladesh is 54.8 per 100,000 people. Stroke results in 888.1 disability-adjusted life years (DALYs) lost per 100,000 [5]. Bangladesh has an estimated population exceeding 170 million, as described by the World Bank in 2023 [6]. It is one of the world’s most densely populated countries, with a reported Gross Domestic Product (GDP) of around USD 437 billion arising from textiles, agriculture and pharmaceuticals, among others [7]. The current GDP per capita is approximately USD 2529, which is relatively low compared to many Western countries, reflecting the low socio-economic health of the country [7]. The capital, Dhaka, has a population exceeding 21 million and is rapidly expanding. Approximately 60% of the population resides in rural areas [8].

This large disparity in wealth creates an apparent urban–rural divide that significantly impacts the nation’s infrastructure. For instance, urban areas have greater access to healthcare with better-equipped facilities and more highly trained health professionals, whereas rural areas face a lack of quality healthcare, leading to issues such as underdiagnosis and, consequently, underreporting of conditions such as stroke. As a result, the reported data on strokes may be underestimated, potentially understating the true prevalence. The prevalence of risk factors varies between rural and urban areas, as well as between the elderly and younger populations [9,10,11]. This reinforces the need to establish a comprehensive data collection system to address stroke and other health concerns across the country.

In order to reduce the impact of this devastating illness, the identification of individuals at risk of stroke and in need of preventive treatment is required. This requires early recognition and a reduction of modifiable risk factors [9]. Proper identification not only reduces stroke incidence but also lessens the burden of stroke by reducing the length of hospital stays and the number of patients with long-term disabilities [10].

For patients who have already had a stroke, appropriate management plays an important role in survival and an eventual return to normal life. Successful stroke management is highly dependent on coordinated care by a multidisciplinary team. In many cases, stroke is a life-changing event which not only has a detrimental impact on the quality of life for survivors but also has negative effects on the affected individual’s family and career as well as on society.

In this review, we will look at the epidemiology of stroke in Bangladesh, how it is treated and the barriers that might exist in how stroke care can be improved in this developing nation.

## 2. Materials and Methods

A systematic search of the PubMed database, Mymensingh Medical Journal, Bangladesh Journal of Neuroscience and Google Scholar was conducted for articles relating to stroke in Bangladesh. Search results meeting the predefined selection criteria included epidemiological studies and reports on the prevalence, incidence, prevention, management and rehabilitation of stroke in Bangladesh. Literature reviews were included if they met the predefined criteria. No restrictions were placed on the selection of articles except for publication in English. Abstracts from the initial selection were further screened to identify relevant articles. Observational, cross-sectional, case-control, comparative, prospective, longitudinal, hospital-based, community-based and population-based studies were included in this review. When the same data were reported in more than one publication or online source, these sources were combined to prevent duplication. Additionally, a grey literature search using searching engines such as Google was performed to identify stroke centres in Bangladesh.

## 3. Results

### 3.1. Incidence and Prevalence

There is currently a lack of well-designed, population-based studies relating to the incidence and prevalence of stroke in Bangladesh, or to the morbidity and mortality rates [2,12]. As a result, limited data exist on the actual prevalence and incidence of stroke in Bangladesh.

The published prevalence data reflect the results of only a few community-based studies (Table 1). According to Mohammad et al. [12], the overall prevalence of stroke in Bangladesh is 300 per 100,000 people, despite variation with age. The male-to-female ratio of stroke is 344:241 per 100,000 individuals, reflecting a 1.25 times higher prevalence in males [12]. This figure is considerably higher than in Western countries, where the incidence of stroke in the USA is around 240 per 100,000 people each year [13], and it is even lower in the UK, with approximately 107 strokes per 100,000 people in 2016 [14]. A recent nationwide study by Mondal et al. [15] found a stroke prevalence of 1139 per 100,000, with the highest prevalence in the Mymensingh division (1471 per 100,000) and the lowest in the Rajshahi division (762 per 100,000). The study is the first-ever nationwide survey in Bangladesh, and it finds that stroke prevalence is significantly higher among males (1362 per 100,000) compared to females (868 per 100,000) [15]. Furthermore, among the male population itself, rural males show a higher stroke prevalence than urban males [12,13].

Stroke in women is poorly reported in Bangladesh, despite being a leading cause of death in females over 60. This is likely a result of cultural factors that affect the reporting of female health statistics in this country [3]. In hospital-based studies, it has been reported that the prevalence of stroke in women is less than in men [18].

Compared to the Western world, a different distribution of stroke types is seen in Bangladesh. In particular, there is a higher proportion of haemorrhagic stroke (HS) (15.7–45%) and cerebral venous thrombosis (CVT). The prevalence of CVT is the highest in South Asia and differs by country in the region but accounts for a significant proportion of strokes in young women in Bangladesh [12,19]. In a hospital-based cross-sectional study by Hossain et al. [9], ischaemic stroke (IS) accounts for 61% of total strokes, and HS accounts for 39% [9]. An observational study by Siddique et al. [19] reports a distribution of 53% IS, 45% intracerebral haemorrhage (ICH) and 2% subarachnoid haemorrhage (SAH) [19]. Interestingly, there appears to be differences in the aetiology of ischaemic strokes, with a greater prevalence of intracranial atherosclerosis compared to extracranial atherosclerosis, which is more common in the Western world.

The higher rate of HS, when compared to Western populations, may result from a preponderance of the research into stroke prevalence in Bangladesh coming from tertiary referral centres. Whilst both ischaemic and haemorrhagic strokes are referred to tertiary centres, HS often requires more specialised care which other centres, such as neurosurgery and critical care units, may not be able to provide. Therefore, a selection bias may be present in the proportion of HS seen in tertiary centres. Although a recent study by Mondal et al. showed more similarity to Western populations, with 79.7% of strokes being ischaemic and 15.7% being haemorrhagic [15], this internal variation further highlights the limitations of Bangladesh’s methods for recording stroke. A poorer control of hypertension compared to Western populations may also explain this difference. The age-standardised prevalence of hypertension is less than 25% in Western Europe in both men and women, compared to 34% in Bangladesh [20]. Hypertension control has improved in Western countries, as evidenced by the Oxford Vascular (OXVASC) study [21]. This hypertension control is unlikely to be the same in developing nations.

The partial data from hospital-based studies indicate a higher prevalence of stroke among the young—especially women—compared to North America and Europe [2]. According to Hossain et al. [22] and Miah et al. [23], the majority of stroke patients are male (52.94% and 56%, respectively) [22,23]. However, a comparative study by Miah et al. [24] reports more strokes in females (58.82%), which supports the results of studies from other South Asian countries [24]. These gender discrepancies may highlight the need for a more accurate and standardised approach to data collection and future study designs in Bangladesh.

### 3.2. Risk Factors

Diabetes, hypertension, smoking tobacco, obesity and high cholesterol levels have all been shown to be significant risk factors in the development of stroke in Bangladeshi populations [9,25,26]. However, identifying patients with these risk factors remains a challenge due to the lack of a common documentation method and guidelines and a significant reliance on the patient history. Hypertension is the most important risk factor in the development of stroke in Bangladesh [15]. Tobacco use in any form is found in 59.6% of men and 28.3% of women in Bangladesh, with 44.4% of men being daily smokers, and a significantly higher prevalence in urban areas [27]. A case-control study conducted in Dhaka, Bangladesh has found that certain risk factors for stroke differ between males and females, highlighting the significant impact of gender on the distribution of stroke risk factors. For males, the significant predictors of stroke include ever smoking (current and former smokers), which is associated with a higher risk, along with diabetes mellitus, hypertension and elevated total cholesterol levels. In females, the significant predictors include diabetes mellitus, hypertension, elevated serum low-density lipoprotein (LDL) and higher serum triglyceride (TG) levels. These findings suggest that the prevention of stroke may need to be tailored differently for men and women based on these risk factors [28].

A study conducted by Sharmin et al. [29] highlights the strong association between smoking and the increased risk of ischaemic stroke. The findings show that 42% of the ischaemic stroke patients are smokers, as opposed to only 11% among the healthy controls. In addition, the duration of smoking is considerably longer in the stroke patients, with a mean of 22.14 ± 10.58 years compared to 4.45 ± 2.58 years in the control group, highlighting the possibility that prolonged smoking markedly increases the likelihood of experiencing an ischaemic stroke [29].

A difference in the risk factors is evident between ischaemic and haemorrhagic stroke, with low total serum cholesterol levels identified as a significant independent risk factor for haemorrhagic stroke [30]. This difference in risk factors is further illustrated in Table 2, highlighting the distinct contributions to ischaemic and haemorrhagic stroke.

In addition to these classical stroke risk factors, non-traditional risk factors have been postulated to contribute to the excess stroke mortality in Bangladesh [31]. According to the results from several studies that have been conducted on non-traditional factors in the Bangladeshi population, these include:

- Squatting position: It has been shown in Indian populations that sitting in a squatting position, which raises the blood pressure about 4–8 mmHg in the central vasculature, may increase your risk of both IS and HS [31]. This is a common posture among the Bangladeshi population due to a traditional sanitary system and may also act as a precipitating factor for stroke.
- Arsenic in drinking water: A study has identified a dose-response relationship between arsenic in tube-well drinking water and stroke mortality. This is particularly the case in women. The results are similar to a study conducted in Taiwan [32].
- Smokeless tobacco: A higher prevalence of stroke is reported in Southeast Asia among women who use chewing tobacco [2].
- Long term betel nut chewing: Betel nut chewing is more common in Bangladeshi males and cigarette smokers. Betel nut chewing is associated with preclinical atherosclerosis due to an altered carotid intima-media thickness [33]. A large population-based cohort study in rural areas of Bangladesh has revealed that 33.2% of the population chews betel nuts [34]. A close association between hypertension and betel quid chewing has also been observed in Bangladeshi females; however, further study is required to determine its role in stroke [34].
- Serum folic acid: Low serum folate concentrations can elevate serum homocysteine. To date, only one study investigating the link between folic acid and stroke in Bangladesh has been published, and a significant association is reported [35].
- Temperature: A positive correlation has been observed between temperatures and IS, which is more common in summer. In contrast, HS is more common in winter [36].
- Hyperuricaemia: A correlation between high uric acid levels and an increased risk of stroke has been observed, including high rates of mortality, in patients with a background of hyperuricaemia. A study by Khalil et al. [37] has found that higher levels of serum uric acid (SUA) are significantly associated with the acute phase of ischemic stroke, indicating that an elevated SUA may be a risk factor for this condition [37]. However, after adjusting for other risk factors, this association is observed only in females, suggesting a potential gender-specific risk factor. Interestingly, several studies in Western populations oppose this view, proposing that uric acid may have neuroprotective effects due to its free radical scavenging properties, potentially reducing neurological deficits post-stroke [38]. The URICO-ICTUS trial assesses the effectiveness of uric acid therapy when used in conjunction with alteplase in 411 patients across Spain. Thirty-nine percent of the patients treated with uric acid achieved excellent outcomes, compared to 33% in the placebo group. Interestingly, there is also an apparent sex-based response, with the therapy showing benefits only in females [39].
- Hypocholesterolemia: A case-control study shows that low cholesterol levels increase the risk of HS by more than six times [30]. Similar findings have been documented in studies conducted in other developing countries in the world, such as India as well as certain parts of Asia [40]. The overall prevalence of dyslipidaemia in an adult Bangladeshi population is recorded as 76.7%, of which 35.7% are reported to have high triglyceride levels and 18.5% have high levels of LDL [41].

**Table 2:** Classical and non-traditional risk factors for stroke associated with populations in Bangladesh [2,42].

Classical Risk Factors		Non-Traditional Risk Factors
Non-Modifiable Risk Factors	Modifiable Risk Factors	
Age	High blood pressure	Water pipe use
Gender (male > female, except in very young and old age)	Heart disease (atrial fibrillation, heart failure, endocarditis)	Desi ghee (saturated fatty acid)
Ethnicity (Afro-Caribbean > Asian > European)	Diabetes mellitus	Smokeless tobacco
Heredity and genetics	Hyperlipidaemia	Infection (Tubercular meningitis)
Previous vascular event (myocardial infarction, stroke or peripheral embolism)	Atherosclerosis	Arsenic in drinking water
Fibromuscular dysplasia	Obesity and physical inactivity	Squatting position during use of the toilet
Right to left shunt caused by patent Foramen Ovale, arterial septal defect, ventricular septal defect and pulmonary arteriovenous malformation	Tobacco use and smoking	Folate deficiency (further studies required to confirm)
	Oral contraceptive pill	Russell viper snake bite
	Polycythaemia	
	Increased plasma homocysteine	
	Excess alcohol consumption	

In the paediatric population, a study by Fatema et al. (2022) [42] has found that among 82 children studied in Bangladesh, 36.5% presented with recurrent strokes, while the remaining experienced their first episode, indicating a significant proportion of recurrence [42]. The primary cause of recurrent stroke is intracranial vasculopathy, particularly vascular narrowing and moyamoya disease (MMD).

### 3.3. Management

As a developing country, management protocols in Bangladesh depend on the availability of an acute stroke service in the emergency room (including urgent CT and tissue-plasminogen activator (tPA) administration) and the availability of a dedicated stroke care unit. However, an acute stroke service is currently found only in tertiary-level hospitals or a few expensive private hospitals in Bangladesh [43]. There are no separate stroke units, and treatment is administered by either a general practitioner or a neurologist [2]. Bangladesh is a country with over 160 million people, and it is believed that there are only 160 neurologists in the whole country [44]. By comparison, in 2022, there were around 1638 full-time neurologists in the UK, which has a population of approximately 68 million [45]. This lack of neurological care for stroke will further impede adequate treatment for the population of the country.

Management guidelines for stroke in Bangladesh have been proposed and are based mainly on guidance from the National Institute for Health and Care Excellence (NICE) in the UK. However, the implementation of such guidance is poor. This may be due to several issues, including staff education, financial limitations, geographical location and infrastructural deficits. This results in a significant variability in the level of stroke care provision at the front line.

### 3.4. Hyper-Acute Stroke Treatment

In Bangladesh, thrombolysis is an exception rather than the rule. Although the use of tPA is indicated in the proposed guidelines, its use is likely to be limited due to the high cost of tPA, socio-cultural factors (patients not presenting in time due to a lack of knowledge about stroke symptoms) and a lack of proper infrastructure allowing people to get to a hospital in a timely manner [46]. There is a lack of comprehensive nationwide data to estimate the annual number of patients with ischaemic stroke treated with tPA or thrombectomy in Bangladesh. However, a meta-analysis across Asian countries reports a utilization rate of 9.1% for the proportion of eligible patients who receive tPA [47]. In a worldwide survey, Asif et al. has found that Bangladesh has the lowest availability to mechanical thrombectomy, at 0.05%, compared to 46.08% in Australia. This is defined as the established proportion of patients with large vessel occlusion receiving mechanical thrombectomy in each region annually [48]. A study by Islam et al. has found that while many patients have a good knowledge of common stroke/TIA risk factors such as hypertension (83.3%) and smoking (85.2%), fewer than 50% recognise atrial fibrillation and carotid stenosis as significant risk factors for the disease [49].

Bhat et al. further emphasises this, illustrating that although 98.2% of participants know stroke is an emergency, and only 26.4% are aware of the critical 4.5 h treatment window [46]. The lack of awareness, combined with misconceptions—such as 80.6% of participants believing that stress is the main risk factor for stroke—overshadows the significance of the actual risk factors of the disease, complicating its early presentation and treatment. The standard hyper-acute ischaemic stroke therapy includes intravenous thrombolysis and mechanical thrombectomy or other methods of recanalization. The use of antiplatelet drugs (such as aspirin or clopidogrel) are also a part of acute treatment. Low molecular weight heparin or oral anticoagulation drugs are generally contraindicated in the acute phase, but they can be considered later, depending on the cause of ischaemia, the need for stent insertion or if the stroke is of cardioembolic origin. In addition, lipid-lowering agents show a positive influence on dyslipidaemia [49].

However, Bhat et al. [50] further demonstrate the issues with knowledge, as only 9.3% of the patients on anticoagulants and 35.2% on antiplatelets know these medications are for stroke/TIA prevention, causing compliance issues [50]. Previous reviews have also contributed to the idea that there is a lack of randomised controlled trial (RCT) evidence for antiplatelet therapy in Asian populations. It is clear that the lack of region-specific data calls for the need for more comprehensive research to assess the efficacy and safety of antiplatelet therapy in South Asian populations, aiding more personalised stroke prevention strategies [51].

Limited evidence is available with regard to decompressive craniectomy and endovascular management. The data are limited to case reports and case series, possibly as a result of the infrequent practice of these techniques in Bangladesh [51]. A prospective observational study in a tertiary centre in Dhaka reports the patient’s financial condition to be a barrier to surgical management in stroke; however, the number of people treated is not reported [52].

### 3.5. Acute Stroke Treatment

Aspiration pneumonia, urinary tract infections, bed sores, convulsions, headaches, insomnia and dysphagia are common complications following stroke.

Deficiencies in the management of complications in Bangladesh often result in deterioration; therefore, the modification of the current practices is required. Insufficient nutrition is a common problem post-stroke in Bangladesh, with 78% of patients receiving nasogastric feeding [53]. This may paradoxically affect nutrition in a country with limited resources, as managing feeding pumps is very resource dependent.

### 3.6. Rehabilitation and Outcomes

In Bangladesh, rehabilitation services are not readily accessible, although such services are available in a few tertiary-level hospitals and private clinical sectors [54]. However, two non-governmental organisations, BRAC (Bangladesh Rural Advancement Committee) and the Centre for the Rehabilitation of the Paralysed, provide long-term stroke rehabilitation services along with primary prevention for those who are unable to afford treatment [2]. Multidisciplinary teams are comprised of physiatrists (rehabilitation physicians), physiotherapists, speech and language therapists, occupational therapists and rehabilitation nurses [54]. Nevertheless, the lack of rehabilitation treatment throughout the country, in both the public and private health sectors, further complicates the management of stroke [55].

Mamin et al. [56] have studied the profile of stroke patients treated in rehabilitation centres in Bangladesh. The majority of the patients attending are educated males under 60 years old (mean age 49 years) living in urban areas (68%). All the patients interviewed state they rely on full-time care from family members, primarily spouses or daughters, placing a significant burden on their caregivers. The average monthly cost of rehabilitation is approximately USD 328, which is over one-fifth of the annual income of the residents in Bangladesh [56]. Without any financial aid from the government, this results in loans and a reliance on family members, causing further stress upon the affected patients.

It is worth noting that the outcomes can be predicted based on the initial presentations and findings. An observational study by Talukder et al. [57] has found a significant number of acute stroke patients without an existing diagnosis of diabetes developed stress-induced hyperglycaemia. These patients have been followed up at discharge and at four weeks and have poorer functional outcomes [57].

### 3.7. Barriers to Stroke Treatment in Bangladesh

Annual health check-up programmes for the age groups at risk are compulsory in many developed nations to identify the traditional risk factors in healthy-looking individuals; the absence of these programmes in Bangladesh may hinder stroke prevention [58]. Furthermore, insufficient numbers of well-trained medico-social workers, named “Shastho Kormi” (health visitors involved in training communities on health issues), in all sectors of society also slow stroke prevention strategies in Bangladesh. Moreover, gender disparity and the persistence of religious and socio-cultural stigmas, such as insufficient attention toward females and hospital phobia, thwart stroke prevention [2].

In the case of stroke intervention, specifically the use of tPA, favourable outcomes have been observed in the developed world, reflecting the availability of acute stroke care [14]. However, as part of the developing world, proposed interventions in Bangladesh are impractical due to infrastructural, socio-cultural and economic deficits. The poor, unreliable infrastructure and insufficient resources of the national health system make the organisation of stroke services nationwide difficult [46].

The inequality in the management practices between Bangladesh and developed countries largely depends on the differences in healthcare infrastructure. In the UK, the NHS is a publicly funded, non-profit, central national healthcare system that offers a comprehensive range of health services for free to the country’s citizens. Meanwhile, the healthcare system in Bangladesh is mostly community-based in structure and offers treatment either for free or for a small amount of money. However, this healthcare system is not centrally governed, is based on primary healthcare concepts and is divided into primary healthcare (Union sub-centres and Thana-health complexes), secondary healthcare (district hospitals) and tertiary healthcare (national-level hospitals), along with private clinics and hospitals [59].

As a result, for stroke patients in developed countries, the process from early recognition to treatment occurs smoothly through the use of a systematic protocol, without consideration of urban versus rural areas or the financial condition of an individual. In contrast, treatment in Bangladesh varies according to geographical and socio-economic status, as the differences in trained physicians, interventions and referrals to higher centres create obstacles to immediate stroke management in rural areas [60]. Bangladesh faces limited access to specialised stroke care, with only 160 trained neurologists serving a population covered by 2213 hospitals and 45,723 registered physicians. The available acute stroke care is situated mainly in Dhaka, comprising two government hospitals as well as five private hospitals, creating a significant geographic disparity. The limited resources for investigative procedures further act as a barrier to acute stroke care, with only 50 CT scanners and 80 MRI machines nationwide [44].

The scenario is different in urban areas due to direct admission into tertiary centres or private clinics, but ignorance of the symptoms of stroke and a low perception of their threat among the public and general practitioners further hinders the implementation of effective interventions [2]. A lack of awareness is a significant hindrance in seeking medical attention. During the COVID-19 pandemic, there was a 46.3% reduction in acute stroke admissions at the National Institute of Neuroscience & Hospital (NINS&H), the largest stroke unit in Bangladesh, between April and June 2020 compared to the first three months of the year. The most pronounced decline was in subarachnoid haemorrhage (SAH) admissions, which dropped by 71.4%. Other stroke types also saw reductions, including ischaemic stroke (45.6%), venous stroke (39.02%) and intracerebral haemorrhage (37.1%) [60]. This decrease in admissions could be attributed to a lack of public health campaigns during the pandemic, which failed to emphasise that medical emergencies such as stroke still require immediate attention despite COVID-19 restrictions. This is particularly important, as an observational study by Hasan et al. mentions that the risk of mortality is five times higher in COVID-positive stroke patients [60]. In addition, an estimate of the average monthly expenditure for a participant’s rehabilitation equates to more than one-fifth of their annual income, reflecting the economic burden of stroke [56]. Regrettably, health insurance coverage, such as the kind available in the US, is not popular in this region, which makes stroke treatment a burden on individuals as well as on the nation [2].

**Figure 1:**
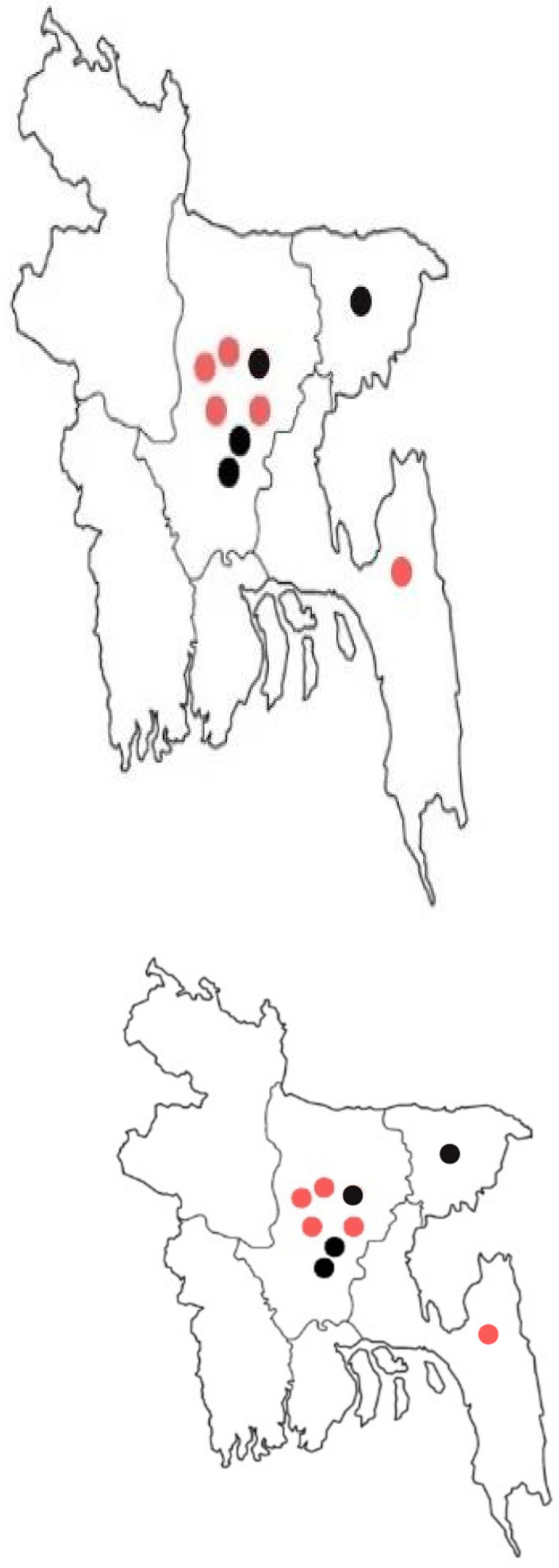
Map of Bangladesh which shows the locations of all the acute stroke services serving a population of over 200 million residents. Red: government hospital, black: private hospital.

Figure 1 and Table 3 display the findings from a grey search conducted to identify the acute stroke centres throughout Bangladesh. The red markers indicate the government-run facilities, while the black markers represent the private institutions. This map reflects the best available information on stroke care locations, illustrating the current access points and highlighting the areas for potential improvement in resource distribution across the country.

## 4. Discussion

Stroke in Bangladesh is a significant health concern, as it is in many nations. The prevalence of the disease is difficult to estimate accurately with currently available research.

HS is more common in Bangladesh than in developed nations, which may be a result of poor hypertension control. The risk factors that affect many Western populations are also very prevalent in the Bangladeshi stroke population, but non-traditional risk factors do exist. These novel risk factors, such as sitting in a squatting position or betel nut chewing, are deeply cultural. Trying to stop the population of Bangladesh from performing these rituals, as a means to reduce stroke rates, could be quite complex. Bangladesh is also limited in its ability to combat stroke throughout the whole country due to the disparity in the services available when comparing the rural and urban communities.

Given that the population is experiencing stroke at an earlier age, implementing targeted social media campaigns on platforms such as WhatsApp and Instagram could be highly influential. These platforms are integral to the daily lives of younger individuals. A recent government survey suggests a total of 179.9 million cellular mobile connections were active in Bangladesh in early 2023 [61]. This makes them effective channels for raising awareness about stroke prevention and early detection. Integrating telemedicine into these strategies could further support outreach by providing accessible medical consultations and timely interventions. However, the success of telemedicine in this context would require significant improvements in imaging infrastructure to ensure accurate remote diagnostics and effective treatment planning.

Improving stroke survival and outcomes in Bangladesh would benefit from a more structured internal health network. The installation of telemedicine services in developed countries has improved the coverage of stroke services, and this may be something that could be applied in Bangladesh. Stroke specialist nurses have improved the delivery of stroke services in countries such as the UK and do not have the associated training costs that doctors do. Employing stroke nurse practitioners may also help to develop a stroke network that could support the widespread implementation of evidence-based practices, including the delivery of thrombolysis.

Ultimately, combating gender stereotypes and having a more organised internal healthcare structure would improve stroke care in Bangladesh. Bangladesh has looked to developed nations, basing its national guidelines on the stroke guidance issued by NICE. International help regarding the organisation of health services and sharing novel ways to treat stroke within a defined budget may help Bangladesh improve its treatment of stroke in the future.

## Figures and Tables

**Table 1:** Summary of demographic data and crude prevalence data from community-based studies.

Study and Design	Location of Study	Year	Sample Size	Crude Prevalence per 100,000	Study Limitations
Mohammad et al. (2011) [12] Stratified random sampling (door-to-door survey)	Community, urban, suburban and rural areas within the Dhaka, Gazipur and Mymensingh districts of Bangladesh	June 2001–May 2003	Urban *n* = 9664	310	- No attempt was made to distinguish IS from ICH. - No neuroimaging was performed. - The decision was made on a purely clinical basis.
			Suburban *n* = 2594	231	
			Rural *n* = 3369	327	
			Total *n* = 15,627	300	
Khanam et al. (2011) [16] Cross- sectional study	Community and rural areas in Matlab, **Bangladesh**	July 2003–March 2004	*n* = 452	900	- Performed as a multi-morbidity study. - Age limitation was ≥60 years. - Diagnoses were based on clinical examination. - Judgements were specific to hemi- or mono-paresis with pseudobulbar syndrome, which can cause overestimation.
Minh et al. (2008) [17] Cross-site study	Southeast Asia including **Vietnam, Bangladesh, Thailand**, **Indonesia** and **India**	2005	*n* = 18,485	500–2000	- Some methodological limitations (Wasay, Khatri and Kaul, 2014) [2]. - Recall bias seen in self-reported prevalence.

**Table 3:** Approximate distances to the nearest stroke centre from cities in Bangladesh, measured from the town centre to the respective stroke centre, using Google Maps to calculate distance to the nearest km.

Town/City	Nearest Stroke Centre	Approximate Distance (km)
Dhaka	Multiple centres within the city, including NINS&H (National Institute of Neurosciences & Hospital	0
Chattogram	Chattogram Medical Hospital	0
Sylhet	Mount Adora Hospital	0
Khulna	National Institute of Neurosciences & Hospital (NINS&H)	130
Rajshahi	National Institute of Neurosciences & Hospital (NINS&H)	245
Mymensingh	National Institute of Neurosciences & Hospital (NINS&H)	120
Barishal	National Institute of Neurosciences & Hospital (NINS&H)	170
Rangpur	National Institute of Neurosciences & Hospital (NINS&H)	310
Comilla	National Institute of Neurosciences & Hospital (NINS&H)	100
Narayanganj	National Institute of Neurosciences & Hospital (NINS&H)	20
